# Brain-Mind Dyad, Human Experience, the Consciousness Tetrad and Lattice of Mental Operations: And Further, The Need to Integrate Knowledge from Diverse Disciplines

**DOI:** 10.4103/0973-1229.77412

**Published:** 2011

**Authors:** Ajai R. Singh, Shakuntala A. Singh

**Affiliations:** * *Editor, Mens Sana Monographs*; ** *Principal and Head, Dept of Philosophy, KG Joshi College of Arts and NG Bedekar College of Commerce, Thane, Maharashtra*

**Keywords:** *Brain*, *Mind*, *Consciousness*, *Western Philosophical theories*, *Mind and Consciousness in Indian Thought*, *Cognitive neurosciences*, *Brain Mind relation*, *Human experience*, *Definition of consciousness*, *Physical changes in brain and mental operations*, *Lattice of mental operations*, *Interdisciplinary work*

## Abstract

Brain, Mind and Consciousness are the research concerns of psychiatrists, psychologists, neurologists, cognitive neuroscientists and philosophers. All of them are working in different and important ways to understand the workings of the brain, the mysteries of the mind and to grasp that elusive concept called consciousness. Although they are all justified in forwarding their respective researches, it is also necessary to integrate these diverse appearing understandings and try and get a comprehensive perspective that is, hopefully, more than the sum of their parts. There is also the need to understand what each one is doing, and by the other, to understand each other’s basic and fundamental ideological and foundational underpinnings. This must be followed by a comprehensive and critical dialogue between the respective disciplines. Moreover, the concept of mind and consciousness in Indian thought needs careful delineation and critical/evidential enquiry to make it internationally relevant. The brain-mind dyad must be understood, with brain as the structural correlate of the mind, and mind as the functional correlate of the brain. To understand human experience, we need a triad of external environment, internal environment and a consciousness that makes sense of both. We need to evolve a consensus on the definition of consciousness, for which a working definition in the form of a Consciousness Tetrad of Default, Aware, Operational and Evolved Consciousness is presented. It is equally necessary to understand the connection between physical changes in the brain and mental operations, and thereby untangle and comprehend the lattice of mental operations. Interdisciplinary work and knowledge sharing, in an atmosphere of healthy give and take of ideas, and with a view to understand the significance of each other’s work, and also to critically evaluate the present corpus of knowledge from these diverse appearing fields, and then carry forward from there in a spirit of cooperative but evidential and critical enquiry – this is the goal for this monograph, and the work to follow.

## I.The Need to Integrate Knowledge from Diverse Disciplines

### Introduction

Concepts related to the brain, mind and consciousness have intrigued both philosophers and scientists since time immemorial. Although the former have speculated on the nature of the mind and put forward many theories of consciousness, the brain as an object of scientific enquiry, and how it relates to functions ordinarily subsumed under the mind, is a relatively recent phenomenon. The emerging body of evidence that the cognitive neurosciences (neurobiology and neurophysiology) and cybernetics are producing cannot but impact philosophers’ understanding of mind and consciousness and compel them to revise many of their long-held theories and convictions. At the same time, many speculative insights of the philosophers regarding mind and consciousness can offer great areas for reflection and experimentation to the neuroscientists.

Philosophy of mind is an active, intensely evolving body of knowledge, as much as are the neurosciences, and one cannot afford to be oblivious of the other.

### The purpose

This 2011 Theme Mens Sana Monograph titled ‘*Brain, Mind and Consciousness: An Interdisciplinary International Perspective*’ is an attempt to present the salient reflections/findings of scientists and philosophers on the interconnections between these concepts and evolve an ongoing dialogue between them so that a robust body of knowledge serves as a foundation for further enquiry in this intriguing, and vastly unexplored, field.

Such a dialogue between the different stakeholders is necessary to evolve a comprehensive corpus of interdisciplinary thought that complements the scientists’ empirical, experimental findings with the philosophers’ reflections, analyses and speculative insights, and vice versa. This is all the more necessary now, as barriers to knowledge sharing need to be replaced by bridges of knowledge diffusion across geographical, cultural, and more importantly, ideological and even disciplinary boundaries. This will bring about much needed integration of thought and understanding in a world which talks of a global village and world unity but is ripped apart by political ideologies and national interests. A topic such as this, which has important inputs from diverse sources, cutting across geographical boundaries, historical epochs and disciplinary bindings, is eminently suited to become one means to evolve such a global outlook.

Hopefully, that is, if it is not asking for too much.

### Contributions of western thought

Of course, as we look back, we can feel satisfied that much has been done in the realm of reflective thought about mind and consciousness down the centuries by some of the great masters of western philosophy [Table 1, see also Table 5 for the great contribution of Indian thought to the subject.]

**Table 1:** Salient contributions of some western thinkers to mind and consciousness

Plato, St. Augustine, Descartes [all three on mind-body dualism; see Plato’s *Phaedo* (many editions), and Descartes on ‘mental substance’ ‘pensee’ or reflexive consciousness, (Descartes, 1644/1911), and Interactionism (Descartes, 1996)] Locke (rejecting ‘mental substance’; see Locke, 1688/1959) Hume (‘bundle concept’; see Hume, 1739/1888) Kant (critique of associationist approaches and stress on ‘phenomenal consciousness’; see Kant, 1787/1929) Berkeley (especially his Subjective Idealism; see Berkeley, 1710/1957) Leibniz (Parallelism; see Leibniz, 1720/1925) Spinoza, Gustav Fechner and W.K. Clifford (Double-Aspect Theories; see, for example, Spinoza, 1985; Clifford, 1879) as also Herbert Spencer and P.F. Strawson (1959) William James (‘stream of consciousness; see James, 1890/1999), Brentano (‘intentionality’; see Brentano, 1874/1924); Cabanis and older masters (Epiphenomenalism; see Cabanis, 1802) Vienna Circle, especially Otto Neurath and Rudolf Carnap (physicalism or extreme materialism; see Carnap *et al*., 1938) Edmund Husserl (Husserl, 1913/1931; 1929/1960), Martin Heidegger (Heidegger, 1927/1962) and Maurice Merleau-Ponty (Merleau-Ponty, 1945/1962)—Phenomenology J.J.C. Smart (Smart, 1959, 1963) and H. Feigl (Feigl, 1958)—Identity theory Russell (‘sensibilia’; see Russell, 1914, 1918, 1921); A.J. Ayer (a type of neutral monism in *Language, Truth and Logic*; see Ayer, 1936) Geulincx and Malebranche (Occasionalism; see Geulincx, 1893; and Malebranche, 1997) Gilbert Ryle (‘the ghost in the machine’ in *The Concept of Mind*; see Ryle, 1949/2000)

This is with regard to the some salient contributions of western philosophers. There will be occasion to review some of their work in this monograph, and the ones to follow.

A lot has been done in the neurosciences by the scientists too (for some salient contributions, see Table 2)

**Table 2:** Some salient contributions of some western scientists to neurobiological research

K.S. Lashley (removal and study of animal brain parts; see Lashley, 1923) H.-L. Tauber (war time brain damage study by EEG and PEG; see Shaffer, 1972) W.G. Penfield (direct stimulation of patient’s brain; see Penfield, 1975) Eric Kandel, Paul Greengard and E. Carlsson (Microstructures necessary for learning, memory and effect of psychoactive substances; Nobel Laureates, 2000; see http://nobelprize.org/nobel_prizes/medicine/laureates/2000/index.html) Paul C. Lauterbur and Peter Mansfield (for their discoveries concerning magnetic resonance imaging, Nobel Laureates 2003; see http://nobelprize.org/nobel_prizes/medicine/laureates/2003) R. Axel and L.B. Buck (genes, protein receptors and odour recognition; Nobel Laureates, 2004;see http://nobelprize.org/nobel_prizes/medicine/laureates/2004)

These, and many others, deserve a close look, which also should be our endeavour in this, and subsequent, monographs.

In the last few decades, there is a vast body of work by different neuroscientists on the neurotransmitters, especially the biogenic amines, aminoacids, neuropeptides, etc., as well as significant advances in neuroimaging studies. There are so many other areas of activity, and the neurosciences are teeming with research work. The emergence of the neurosciences as a distinctive discipline, with inputs from both scientists and philosophers, is an exciting development, which holds much promise and must be welcomed with great joy.

All these shall be reviewed in this, and future, work in this series.

### The need to integrate

Although scientists and philosophers are busy forwarding their respective approaches to brain, mind and consciousness, and with ample justification, precious little is being done to integrate the vast body of knowledge that already exists about these concepts in these independently progressing branches of philosophical thought and scientific experimentation.

The neurosciences are a brave attempt to bridge the gap, but most mainstream philosophers and scientists, much to the dismay of those who wish for greater contact, remain wary of each other. This, of course, is also because both sides carry enormous baggages of past disconnected bodies of work, and present lack of understanding of each other’s thrusts and motivations.

This monograph is a step to help the process of such integration and help develop a comprehensive picture.

### Mind and consciousness

A huge mass of work already exists on the mind. We need to present and review classical and modern concepts and theories about mind and consciousness, including the mind-body or body-mind problem; the idealist and materialist views about mind; the identity, the computational and double aspect theories of mind; monistic and dualistic theories of mind; as also interactionism, epiphenomenalism, structuralism, reductionism, materialism, occasionalism, neutral monism, functionalism, psychophysical parallelism and so many other ’isms.’ No new theorising can afford to ignore this vast corpus of work that has already been bequeathed us by the great theorists of yester years.

The philosophy of mind is intimately connected with the philosophy of action. Therefore, concepts like free will, motive, intentions, cognition, volition, feelings and also ethical issues related to these, are of abiding interest, and therefore of concern for this, and subsequent, monographs in this series. Questions related to cognition like perception, sensation, insight, intuition, judgement, as also thought, analysis, and the notions of doubt, inference, reasoning, logical thinking, and how these are connected to our understanding of the mind and its connectedness with evidences from research in the neurosciences, will also be of interest in this connection.

The problem of consciousness needs to be connected with that of the mind, but not only our philosophical understanding of the mind, which of course is very important, but also the emerging evidence from brain research, especially neuroimaging. Moreover, the various metaphysical positions like the dualist and physicalist theories, and the specific ones like higher-order, representational, cognitive, neural and quantum theories, need to be put in perspective to understand where we stand in our grasp of this complex topic. Also worthy of detailed interdisciplinary analysis are concepts like Qualia, Introspection, Self-knowledge, Creature and State Consciousness, and the mechanism of mental operations [Table 3].

**Table 3:** Some concepts related to consciousness for detailed interdisciplinary analysis

Qualia [the ‘what it is like’ character of mental states; C.I. Lewis, (Lewis, 1929/1956) first used the term in its modern connotation and F.C. Jackson (Jackson, 1982) further defined it; see also P.M.Churchland (Churchland, 1985)] Introspection [including the works of the champions of the introspective method, as seen in the work of Wilhelm Wundt (Wundt, 1897), and Hermann von Helmholtz, William James and Alfred Titchener]; and Self-knowledge, as aspects of consciousness Creature consciousness and state consciousness, as also the ‘state of consciousness.’ Delineating the relation between brain and mind [Tables 6 and 7] and defining consciousness [Table 8]. Detailing the relationship between observable mental operations and related subtle brain physical activity; in other words, how do mental operations result [Figures 6–15]

Work in scientific psychology, especially Behaviourism (Watson, 1924; Skinner, 1953), Gestalt psychology (Köhler, 1929; Köffka, 1935) and, more recently, cognitive psychology with emphasis on modelling internal mental processes and information processing (Neisser, 1965; Gardiner, 1985) also need critical appraisal. A major resurgence of scientific and philosophical research into the nature and basis of consciousness in the 1980s and 1990s, with the works of Baars, 1988; Dennett, 1991; Penrose 1989, 1994; Crick 1994; Lycan 1987, 1996; Chalmers 1996, need to be extensively critiqued as part of this, and subsequent, monographs.

Also noteworthy is the emergence of specialty journals and societies devoted to the interdisciplinary study of consciousness [see, for example, Table 4]

**Table 4:** Some journals/societies devoted to consciousness studies

The Journal of Consciousness Studies, (Available at http://www.imprint.co.uk/jcs.html); Consciousness and Cognition, (Available at http://www.elsevier.com/wps/find/ journaldescription.cws_home/622810/description#description); Journal Psyche, (Available at http://www.theassc.org/journal_psyche) Also, professional societies like Association for the Scientific Study of Consciousness – ASSC. (Available at http://www.theassc.org).

All these are exciting developments that need to be noted here and their role and influence on present and future developments in the field closely studied, and furthered.

### Concept of mind in Indian thought

There is a vast, and largely unexplored, mass of writings related to the concept of mind in Indian thought (for an overview, see, for example, Chennakesavan, 1991), which needs a careful and detailed exposition and evaluation. By unexplored, we mean at the international level; at the Indian level, a lot of work is being done, which, however, has still to receive international attention. That is probably because most of it is at the local level, in the local idiom, and largely explicative, often idolatry. The way forward is to yield way to robust evidential and sharply critical enquiry of even the most hallowed concepts in the tradition so as to establish their contemporary relevance. The greatest of masters, the greatest of treatises and the most revered of concepts must be subject to such a scrutiny.

First of all, let us see what these are. Concepts related to mind and consciousness have occupied Indian thinkers for centuries. Some (like the ones in Table 5) need to be comprehensively critiqued, and this shall be the task for the present and future issues of *MSM*.

**Table 5:** Some topics for study based on concepts of mind, consciousness and brain in Indian thought

Topics*
Concept of Mind and Consciousness in the Indian Philosophies: An Overview Relevance of Indian Concept of Mind and Consciousness to World Philosophy Analytical study of the concept of Mind in the Indian philosophies Comparative study of Mind in Indian and Western thought Mind in the different *darśanas* Mind in the *Upani* 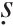 *ads* Is Indian Thought on Mind and Consciousness Relevant Today? Jaina concept of Mind and Consciousness Mind and Consciousness in *Carvāka* thought Nyāya concept of Mind and Consciousness Mind and Consciousness according to Sri Aurobindo Mind and Consciousness for Rabindranath Tagore Phenomenal reality (*prā* 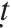 *ibhāsika-sattā*), empirical reality (*vyāvahārika-sattā*), and absolute reality (*pāramārthika-sattā*) *Vedānta*, Mind and Consciousness Transcendental consciousness as “one only without a second” (*ekameva advitīyam*). Advaitic concept of mind and consciousness Buddhist concept of mind and consciousness Samkhya concept of mind and consaciousness Mind and consciousness for Swami Vivekananda Mind, Consciousness and Sri Krishnamurti Gandhi on Man, God and Consciousness Modern Indian Thinkers on Mind and Consciousness K.C. Bhattacharya and S. Radhakrishnan on Mind and Consciousness Mind and consciousness for Acharya Rajneesh Mind and Consciousness in Indian Thought of last two decades 1990-2010. The Future of Indian Thought on Mind and Consciousness Mind and Consciousness in the *Brahma-sūtra* of *Bādarāya* 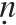 *a* The state of *Sthitapragña* Mind and Self in Indian thought *Prājña* of the deep-sleep state, *Taijasa* of the dream state, *Viśva* of the waking state Self above matter *Tajjalān* and *kalpita* Brahman and *Ātman* Ego (*aham*) and *cidābhāsa*, i.e. consciousness reflected in the internal organ Mind not identifiable with Self according to Indian thought *Gau* 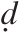 *apāda’s* declaration, *“upadeśād-aya* 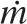 vāda 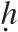 ” and *“jñāte dvai* 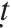 *a* 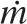 na vidyate” Brahman/Ātman neither immanent nor transcendent Brahman/Ātman both immanent and transcendent Empirical-relational objects with class feature (*jāti*), quality (*gu* 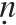 *a*), action (*kriyā*), or relation (*sambandha*), and signified by a conventional word (*rū* 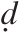 *hi*) The knower (*pramātā*), and the Self Negative scriptural concepts like *“neti neti”* Secular and sacred *śabda* Ultimate reality trans-empirical and trans-relational *Anta* 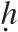 *kara* 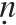 *a* as internal sense organ The concept of *manas* *Jiva, manas* and *ātman* *Vasanā, vairāgya* and *manas* The state of *sat-cit-ānanda* Knower (*jñātā*), “I” (*aham*) and “this” (*idam*). Witness-consciousness (*sāk* 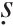 *i-caitanya*), *Pramā* 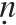 *a* and *apramā* 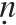 *a* Distinguishing valid cognition *(pramā) from erroneous* (*ābhāsa-jñāna)* Consciousness as self-established *(svatassiddha)* and self-luminous and the transcendental a *priori* *Upani* 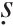 *adic* theory of three worlds Human being as material *(ja* 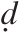 *a)* excepting the Self or Consciousness Mind a sentient entity carrying the reflection *(pratibimba)* or semblance *(ābhāsa)* of Consciousness The five organs of perception, the five organs of action *[karmendriyas]*, the five vital breaths *[pra* 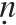 *as]* The mind *[manas]*, intellect *[buddhi]*, egoity *[ahamkāra]* and the mind-stuff *[citta]* Waking experience *(jāgrat)*, the world of dream experience *(svapna)*, and the world of deep sleep experience *(su* 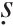 *upti)* *Upani* 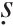 *adic* tradition and the Fourth *(caturtha)* beyond the three worlds in item 59. Consciousness *(cit)* and experience *(anubhava)* *Viśva, Taijasa* and *Prājña* Triple Stream of Experience *(avasthā-traya)* “I” as knower *(jñātā)*, as doer *(kartā)*, as experiencer *(bhoktā)* *Jiva* and its *kośas* The *Kośas: Annamaya* [sheath of food and matter], *pra* 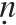 *amaya* [sheath of vital breath], *manomaya* [mental sheath], *vijñānamaya* [intellectual sheath] and *ānandamaya* [the sheath of bliss], and what do they signify in understanding the Self Mind empowered with cognition of other objects, sense of “I” and “mine”, and also selfconscious when need arises Self-conscious mind and *jīva* Self or foundational Consciousness Self and the Mind Śa 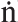 kara and *jñāna-karma-adhikāra* Consciousness as support *(adhi* 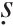 *thāna)* of objects of the entire world Advaita *Vedānta* characterised as “transcendental phenomenology” and “metaphysics of experience” Advaita as both pluralistic and monistic *Citta* and *samskāras* *Buddhi, ahamkāra* and *citta* *Patanjali Yoga* and the eight fold path Buddha’s four noble truths and eight fold path *Citta-v* 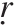 *tti-nirodha*: how does it relate to the concept of Mind in Indian thought *Citta* and *v* 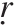 *itts* *Ahamkāra* [or egoism] and the Mind The state of mindlessness The state of *mok* 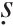 *a* *Kaivalya, Nirva* 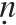 *a, Apavarga, Nihśreyasa* The concept of liberation in the Indian philosophies *Ātman* and the Mind Configuration (*avasthā*), place *(deśa)*, time *(kāla)*, and qualities *(gu* 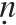 *a)* The concept of brain in Indian thought Ayurveda, mind and brain Body represented by the brain, mind represented by *vijñāna* and *ātman* represented by the life principle as making for the complete man The state of *savikalpaka* and *nirvikalpaka samādhi* The *Gu* 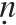 *as* - *Sattva, rajas, tamas* - and the Self Advaita as affirming monism without denying pluralism *Nai* 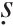 *karmya-siddhi of Sureśvara.* *Buddhi* or cognition The concept of *Citta* The concept of *d* 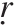 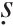 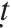 *i* The *Indriyas, Karmendriyas*, and *Jñānendriyas* *Jñāna* or knowledge *Sm* 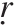 *ti* or memory Absolute Consciousness or *turīya* Mind as an internal organ of sense Mind as self Mind as not the self Mind as minute and subtle Mind as instrument of knowledge Mind as instrument of the soul Self-cognition of Mind Mind as cause Mind and dream experience Mind as reduced to a machine Sense organs and mind contact *Vrtti* or mental mode Self or *Ātman* or Soul Self as pure consciousness *Vijñāna* or discrimination *Prajñā* or intelligence *Sannikar* 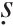 *a*, or relation between mind, sense-organ and the object *Samkalpa* or power of conception.

Table 5 is a representative sample. Just a perusal of the topics will make clear the amount of work available, and the quantum of work now to be done over it, to make it a corpus of contemporary international relevance. As noted earlier, for internationality, critical and evidential enquiry, not necessarily only reverential and explicatory, will need to be seriously forwarded by those who have the interest of Indian thought at heart.

We appeal to all scholars of Indian thought to tackle these, and related topics, in future issues of the *Mens Sana Monographs*; but always remembering that they present their ideas in a modern idiom (that is, forwarding the critical and evidential approach, we talked of in the earlier paragraph, which hopefully can also be comprehensive and eclectic).

For this, it is first of all necessary that contemporary scholars/researchers of Indian thought agree on definitions of concepts/terms, and also agree to a Standard English translation of Sanskrit terms, as also of original and secondary treatises. This is a huge intellectual task, for which a group of recognised authorities in the respective *darśanas* will have to get together and evolve a consensus on the definition of terms, on the authenticity of their translation, and on the authentic translation of the important treatises of their respective *darśanas*. A ten-year period must be devoted exclusively to this enterprise, if not more. This is one task the Indian Council of Philosophical Research, and such other Bodies, which have the interests of the Indian Philosophies at heart, need to further. For we need not just proliferation of Indian thought, but also its orderly progression. Further explication and critical evaluation will be greatly facilitated by this initial ground-work.

### The brain

The brain is a complex organ, the structural correlate of the mind, centre and head of the central nervous and neuroendocrine systems, whose various areas are yielding fascinating, though rather tardy, information to science and biology. Areas like the cerebrum, which controls higher functions like thought, language, moral and social conduct, creativity, spirituality etc, need as much study as the limbic system connected with emotions and sexuality; and the neuroendocrine system which controls an organism’s response to stress, emotions, thoughts and feelings. As also the various pathological conditions that result from toxic, metabolic, infectious, degenerative and congenital/traumatic conditions of brain pathology; not to forget the great number of neuropsychiatric conditions with hitherto ill-defined aetiology like schizophrenia, the affective disorders, epilepsies, dementias and the various neuroses that are great areas of interest and activity in clinical and research psychiatry/neurology.

The emerging vast body of evidential findings from the various neurosciences, including classical psychiatry/neurology, neurobiology, neuropsychology and neurophysiology, needs a thorough presentation and a close look if present and future philosophic theorising has to be grounded on solid foundations. The interdisciplinary field of cognitive neuroscience, which connects the sciences of the brain [neurosciences] with the sciences of the mind [cognitive science], needs a special and careful look. Neuroimaging and ionic/molecular processes studies are yielding fascinating information of brain function that philosophers of mind can ill afford to ignore. The papers of neuroscientists and a close look at their findings will be a special feature of this monograph, and the ones to follow.

Also worth a close look is the need to dispel confusion about the concepts ‘brain’ and ‘mind,’ and their relationship. Centuries of writings and opinions have only served to blur margins and make vagueness the hallmark of the relation between these two concepts. In general, historically, the concept ‘mind’ has received greater attention than the entity ‘brain,’ and that has not helped clear the confusion. The reason most probably was that although brain activity may have fascinated mankind down the ages, for want of precise instruments to measure it, reflection in the form of enquiries into a functional entity called ‘mind’ gained precedence over evidential enquiry into a structural entity called the ‘brain.’ Hence, the concept ‘mind’ gained greater prominence over the entity ‘brain.’ This is in urgent need of repair, of course without overbalancing to consider structure to be all, and neglecting function, which is what really is of importance.

In no theories of the philosophy of mind of earlier times, both in the east and the west, was there any significant attention given to the entity called brain. Nor have modern philosophical theories done much to repair the damage. That we have continued with this neglect is obvious from the fact that there is no entry ‘brain’ in any dictionary (Flew, 1983; Lacey, 1986; Frolov, 1984) of philosophy, or any encyclopaedia for that matter, either print (e.g. Edwards, 1972) or online (e.g. Stanford, 2010; Internet Encyclopedia of Philosophy, 2010). We had access to these; others, or more recent editions, may have hopefully set the damage right. This is in urgent need for repair. How can any discipline theorise effectively about the mind without understanding what is the brain?* Mercifully, a dictionary of psychiatry did not miss having an entry on mind, while of course having one on the brain [Campbell, 2004; brain on p94; mind on p408], probably because the discipline can ill afford to neglect either.

**Note:**

*[A.S. adds]. I wish to share my dismay here. I find it intriguing, if not downright appalling and preposterous, that a group of people that makes the greatest use of a structure also simultaneously makes the greatest attempt to say nothing about it in their works. That group is the group of philosophers, and that structure is the brain. No dictionary, no encyclopaedia of philosophy, and no author worth the name in mainstream philosophy, has condescended to provide even a simple working verbal sketch of the human brain, obsessed as they all are with providing the most detailed and intricate theories of its functions (as the mind). In perpetuating this fallacious state of affairs, use of the term mind in place of the term brain maybe responsible, of course. But, in general, they have shown indifference at best, and have tried their utmost to remain ignorant all through the centuries; even today, in the vast majority of cases. This is denial at its worst. I use harsh words, but am left with no option. The intention of course is not to castigate, but to awaken the philosophers from their dogmatic slumbers. They need to study the structure that is the very *raison de etre* of their existence.

In sum, then, neglect of the entity brain by philosophers of the past is in urgent need of repair by the philosophers of today. Of course those doing interdisciplinary work have tried to repair the damage, but there have to be more shoulders to the wheel.

An important step in this direction is to summarise the following two intellectual tasks that beckon us today:

1. Avoid neglect of the entity brain by philosophers of mind.
2. Avoid use of the term ‘mind’ when what is correct is the term ‘brain.’

A significant initiative in this direction would be to clarify the connection between ‘brain’ and ‘mind,’ and try and evolve a comprehensive definition of consciousness. These shall be our concern in the next two sections [II.1, II.3]. Also intimately connected with these is the mechanism of mental operations, which we shall detail in the section to follow them [II.4].

## II. Brain-Mind Relationship, Human Experience, Defining Consciousness, and the Lattice of Mental Operations

### II. 1. The Brain-Mind Dyad: And a formulation to tear apart, if you can

**Figure 1:**
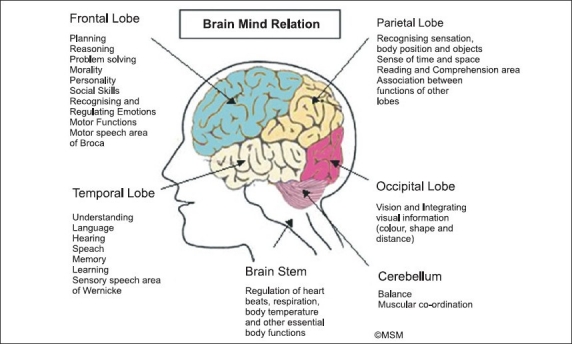
The structure Brain carries out functions like thinking, emotions, problem solving, sum total of a person’s personality including moral standards/judgements/reasoning etc, language/speech, hearing, vision, making sense of perceptions and regulating motor activities, balance/coordination, heartbeat/respiration/other vital functions, hormonal and related balances. All these functions can be subsumed under a broad category of functions called Mind. Brain is the structure, Mind a collection of its functions. Brain and Mind, though connected concepts, are not synonyms. They should not be used interchangeably.

**Figure 2:**
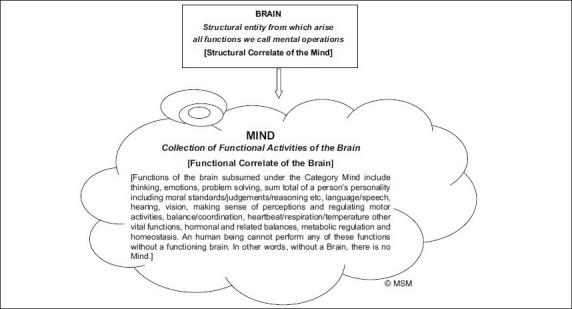
The structural entity from which all mental operations arise is the brain. The collection of these functional activities of the brain is called the Mind. Brain is the structural correlate of the Mind. Mind is the functional correlate of the Brain. Brain is a structural entity, Mind is not a structural entity: it is a convenient label for a collection of Brain’s activity. One should avoid reifying the Mind.

In this connection, we put forward a formulation [Figures 1 and 2, Tables 6 and 7] that delineates the relation between ‘brain’ and ‘mind’ [Figures 1 and 2, Table 6] and between ‘brain,’ ‘mind’ and ‘consciousness’ [Table 7], to accept, modify or criticise, and to tear apart, if you can.

**Table 6:** Brain, mind and their relationship

Brain and Mind, though related terms, are not synonyms. Therefore, they should not to be used interchangeably. Mind is the functional correlate of the brain. Brain is the structural correlate of the mind. In other words, brain is the structure, mind its function. Like eye is the structure, sight its function. Although, metaphorically speaking, they are two sides of the same coin, we must remember that one is an entity [brain], other its operation [mind]. We neglect accepting this at our own peril. Mind is not equal to brain. Mind = Brain functions. Is then Brain = Mind structure? No. Brain= Physical Structure from which Mind originates. Mind, as a collection of brain functions, has its own internal functional structure, which is the result of brain operations. Mind is the product of brain activities. It is the brain and nervous system which run the rest of the body and all its activities, including thinking and action in all their forms, not the mind. Mind is just the sum total of all brain functions. We must avoid use of sentences like, ‘My mind is not working’; ‘What the mind cannot think, the eyes cannot see’; ‘Thinking is a function of the mind’ etc. What we should rather say is, ‘My brain is not working’; ‘What the brain cannot think, the eyes cannot see’; ‘Thinking is a function of the brain.’ We must similarly avoid use of terms ‘conscious mind,’ ‘unconscious mind’ etc. What we mean actually is ‘conscious brain’ and ‘unconscious brain.’ This reification of the operation ‘mind’ must end, and be replaced by the entity ‘brain.’ It may sound odd to say this for a while, for it has behind it centuries of habit. But habits, which obfuscate issues, need to be forsaken. What we call, ‘I’, or self-identity, is itself the product of the brain. And therefore, one of the brain functions to be subsumed under the category mind. Why so? Just let the person be brain dead, and where is his ‘I’ sense? Similarly, we must accept that mind is the product of the brain. Just let the person be brain dead, and where is his mind? Our metaphysical understanding of the self as transcendental, immanent, non-material etc is itself the product of our brains. Let there be the no brain, and can we think of these concepts? Whether such a self exists is in the realm of reflection and speculation, which themselves are products of the brain. No metaphysician, howsoever revered or hallowed, could have produced his grandest formulations bereft of his brain, a structure singularly neglected through the centuries by philosophers and theorisers. Brain is an entity that exists, mind is a concept we have formulated for our understanding of its functions. Brain is the producer, mind its product. Without a brain, there is no mind.

The further ramifications of the formulation in Table 6 may also interest us here. We must clarify the existence of, and connection between, brain, mind and consciousness [Table 7].

**Table 7:** The relationship between brain, mind and consciousness

What then exists: the brain, the mind, consciousness? The further formulation is as follows: The brain exists as a structural entity. The functions of the brain, called mind, exists as its activities. (Which means the brain doesn’t exist as an activity, and the functions of the brain do not exist as structural entities) One of these functions is consciousness. Which means it is a function of the brain, and part of the collection of activities called mind. The mind does not exist as a structural entity in the human body, like the brain does. It is just a convenient label for all the different activities of the structural entity called the brain. The essential characteristic of the brain is that it is the head or centre of the human body where perceptions from the outside world (and from the organism itself) are received; and executive orders to the different areas of the body (or parts of the brain itself) are sent. It is also the centre for all thought, emotions, morality, aesthetics, will, self-identity, actions etc. Everything ever thought of, acted upon or written about is a product of some brain somewhere. This activity is what is subsumed under the concept ‘mind.’ The major problem in philosophy has been to neglect the entity called ‘brain’ by giving thestatus of a structural entity to the ‘mind.’ In other words, ‘mind’ as a functional concept has usurped the structural entity status of ‘brain.’ That has given rise to the huge confusion between the mind and brain, only matching the confusion between mind and body. Can human beings, or other organisms that have a brain, carry out any mental operations without consciousness? And by consciousness, we do not mean only the waking state, for there are states of consciousness, including coma. The answer is no. Which means without one of the states of consciousness, no mental operation is possible, whether in the waking, sleeping or comatose/delirious state. For mental operations occur in all these states. They stop only with the death of an organism. Can human beings carry out any mental operations without a brain? Without a brain, no mental function is possible, unless it is replaced by an ‘artificial or surrogate brain.’ But even if we could keep the person alive, say by a heart-lung machine/ventilator, when the person is comatose, for example, any mental operations that can be detected in such an individual [by means of EEG or fMRI etc now available] cannot be possible without a functioning brain, howsoever minimal the functioning may be. Even if we were to ever develop a mechanical apparatus which carries out brain functions in a living organism (e.g. in the brain dead), it would still be of the nature and function of the living brain, and would become a ‘mechanical brain’ or ‘surrogate brain.’ As regards the existence of Pythagorean theorem even after Pythagoras’ brain has ceased to function, let’s not forget Pythagorean theorem is a thought, and a functional output of his brain. A functional output can definitely exist even after the structure that produced it is no longer in existence. It has its own independent existence, here as a thought, which can be then understood and accepted/rejected by other brains. The point was—could human beings, who are alive, carry out any mental functions, without a brain. Could Pythagoras have thought of his theorem without his brain? Could we understand Pythagoras’ theorem today, or ever, without our brains? The answer has to be a no. Can someone refute this? Can we say further that the mental operation Pythagoras created (or discovered) survives his death? Yes, that is true, but that is because the mental operation survives as a ‘product’ in the form of a thought. Any product can survive after the ‘machine’ that produced it no longer exists. A machine produces a fan. After it is produced, the machine is not needed for the further existence of the fan. A child is produced by a mother. For further survival, the mother is not essential. The thought, after being created, develops its own independent existence. However, human thought cannot be created without a brain. At least until now. And the thought, after its creation, cannot be understood later by another, without the presence of a brain in him. Hence to the question: Can human beings carry out any mental operations without a brain? The answer has to continue to remain a no. It may be convenient to label this formulation as supporting or falling under one of the ‘isms’: epiphenomenalism, physicalism, materialism, central state materialism, identity theory, brain process theory, double-aspect theory or monism or some admixture of these. But that may be facile, and a convenient method of categorising and getting done with a formulation, which is hardly the purpose here. It is not to forward any ‘ism’ that we present this. It is to put forward a formulation that any follower of any ‘ism’ can refute, if possible, or accept/modify, and thus help present a picture that helps clear the confusion, and take thought forward there-from.

### II. 2. The human experience triad

**Figure 3:**
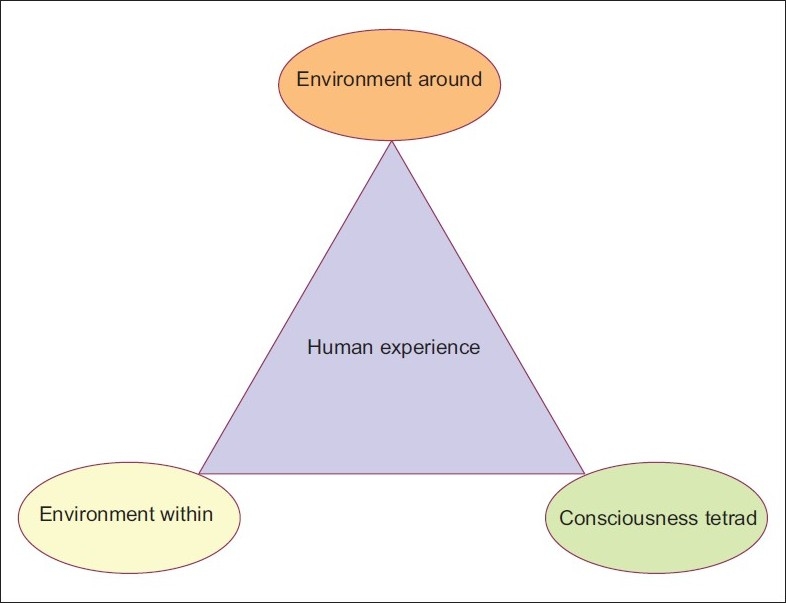
A living human being’s life experiences are a dynamic interaction between the environment around, the environment within, and the different forms of consciousness [Consciousness Tetrad, see Figures 4–5] that help him make sense of both these environments. What is applicable to human beings is applicable, to a lesser extent, to other living beings too, taking for granted that they also can experience, and humans are the most evolved of living beings, as yet.

All living human experience is a dynamic interaction of a triad of inputs [Figure 3]:

1. From the environment around–nature [living, non-living], people and their products, and our perceptions and interactions with them.
2. From the environment within–perceptions/sensations/cognitions of internal bodily processes–body includes the rest of the body *and* also the brain.
3. A consciousness that makes sense/nonsense of these two environments.

It is consciousness, then, that can guide or misguide us in our experiences. Hence, it becomes essential to study its structure and function all the more carefully.

### II. 3. The consciousness tetrad (CT)

**Figure 4:**
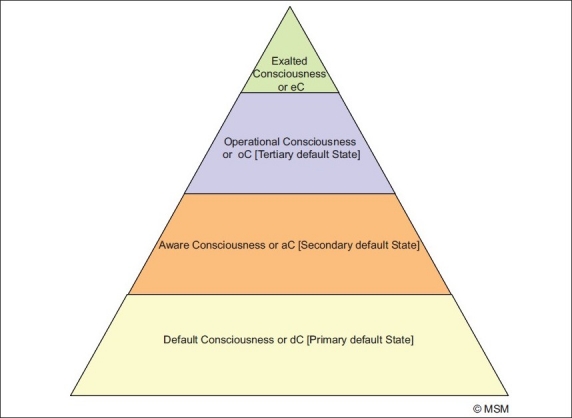
All forms of Consciousness, Aware, Operational and Exalted, rest on the primary default state, dC, which is their base. Meaning without it, the other states cannot arise. From this base arises the aware state, aC, in which we are awake, drowsy, in sleep, or in an altered state eg hallucinating, delirious etc. This is the secondary default base, meaning States 3 and 4 rest on it and cannot result without it. This aware state is the base for the operational state, oC, wherein arise thought, emotion, morality, aesthetics, creativity, motor and sensory operations etc. This is the tertiary default state, meaning State 4 rests on it and cannot result without it. This operational state, used with discretion, results in the exalted state, eC, wherein one can commune with, meditate upon, the divine, Self, God, Brahman etc; it also results in some special forms of creativity (meditative or spiritual creativity).

**Figure 5:**
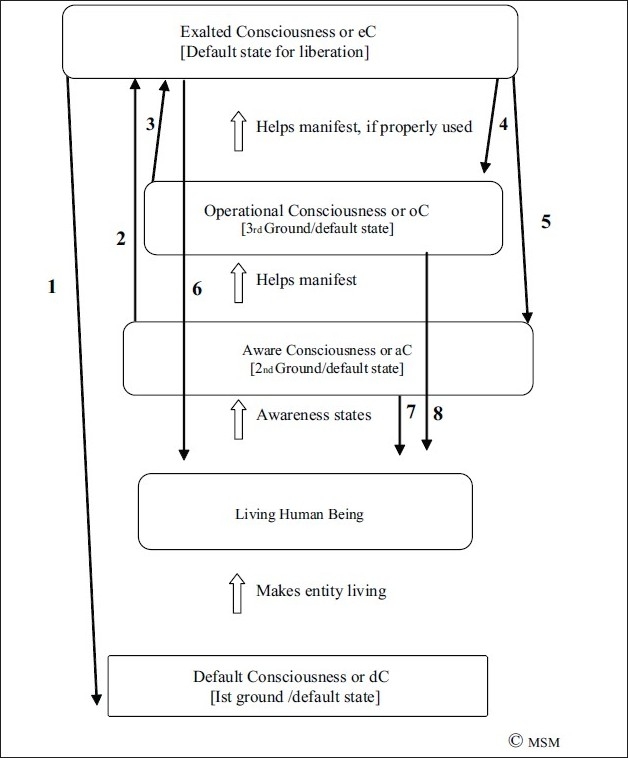
It is default Consciousness, dC, the first ground state, which makes a human being living. Having life, arise the awareness states, aC, which become the 2^nd^ ground state for brain activities to start. Upon this state operate our thoughts, feelings, actions and sensations etc., oC. From this state, under proper conditions e.g. contemplation, meditation, introspection, prayer etc, or a combination of these [all functions of oC], arises the exalted state, eC. The eC tries to cut off all oC and reach the dC state, devoid of all thought, sensation and feelings etc [Arrow 1]. Both aC and oC try to impinge on eC [Arrows 2,3], and eC tries to also regulate and alter oC and aC [Arrows 4,5,]. eC, oC, and aC, all in diverse ways, operate on the Human Being [Arrows, 6,7,8]. This is the dynamic manner in which a living human being is regulated by his various forms of Consciousness, and in turn regulates it.

The most contentious issue in the study of consciousness is to define it. But before we think of defining, we must find out how differently we can view consciousness. At least the following four important considerations must be given serious thought [Table 8, and Figures 4 and 5]: the Tetrad of *Default Consciousness, Aware Consciousness, Operational Consciousness and Exalted Consciousness.*

**Table 8:** The consciousness tetrad [CT]: Four important forms of consciousness: default, aware, operational and exalted

Default Consciousness, dConsciousness or simply dC: Consciousness as a default state that separates the living from the dead, the living from the non-living. This is the ground state on which the figure of 2^nd^, 3^rd^ and 4^th^ states rest. It is like the blue screen of the TV set or computer, which you see when the set is just put on. This is of concern to general medicine, law and society in general, for it helps differentiate the living from the dead. Its absence results in a flat EEG record. Aware Consciousness, aConsciousness or simply aC: Consciousness as awareness which ranges from the awake state through drowsy state and sleep (REM and NREM); as also altered states like semiconscious, unconscious, delirious, comatose etc. This is of concern to general medicine, psychology and neuropsychiatry/neuroscience. Its presence is in the form of different waves in an EEG record, for example, alpha, beta, gamma and delta, and their admixture and due/undue prominence. Operational Consciousness, oConsciousness or simply oC: This is consciousness as sensory, motor, cognitive, conative, emotive, aesthetic, ethical, creative etc abilities. Awareness of mental operations (i.e., A [oC]) is itself a form of operational consciousness. oC is best studied by psychological tests and functional neuroimaging studies. It results in alpha and beta waves on an EEG record. It is of concern as much to cognitive science as to philosophy of mind. Exalted Consciousness, eConsciousness or simply eC: Consciousness as an exalted state of connecting with the divine, soul, inner self, God, special forms of creativity (spiritual), meditation etc. This is of major concern to metaphysicians, who often consider the term ‘consciousness’ to be synonymous with this exalted consciousness, and therefore find the other three ways of looking at consciousness inadequate. In recent years, eConsciousness has also become interesting to neuroscience as an object of study. The EEG record is alpha and theta waves. It is being probed and studied, though rather inadequately, by functional neuroimaging and other means. The obvious hindrance being lack of subjects, mainly due to lack of faith in the scientific method in the practitioners of eC.

Although stakeholders appear different [Table 8], all these forms of consciousness are actually inter-related and interdependent. The picture, however, is so obfuscated that often the only thing common between scientists and philosophers is use of the word ‘consciousness’--so different are their interpretations of the term. Their diverse understandings have added as much colour as confusion to this hitherto ephemeral concept. It is time it is retrieved from the realms of speculation and rhetoric to the world of empirical and evidence-based study. Hence, the attempt below.

First and foremost, it is necessary to understand the four different forms of consciousness [Table 8] and understand the rough structure of this tetrad [Figures 4 and 5]. This will help understand the dynamics of their relationship with each other and the living human in whom they interact [Figure 3]. Thus, it will develop a comprehensive picture of the living human being’s triad of influences-a being in dynamic interaction with his internal/external environment, and the CT that makes it all possible [Figure 3].

### A brief explanation of CT

Default consciousness, dConsciousness or simply dC; Aware consciousness, aConsciousness or simply aC; Operational Consciousness, oConsciousness, or simply oC; and Exalted Consciousness, eConsciousness, or simply eC are the four forms of the CT that must occupy our attention [Table 8, Figures 4 and 5].

*d*C comes first in life, and is the last to go too [Figure 5]. It is essential for the other three states to manifest. *a*C regulates both *o*C and *e*C that follow. Most of our routine life activities depend on aC on whose ground the figure of oC functions. *o*C can help, but often hinders *e*C. And *e*C is dependent on, but often needs to give up any but the bare minimum of *o*C and *a*C. In its most pure form, *e*C links to *d*C alone. In fact, *e*C may become *d*C [Figure 5]. A living human is regulated by, and in turn regulates, the triad of the environment around, the environment within, and this tetrad of consciousness that helps him make sense of both [Figure 3]. [Please also go through the legends to the Figures 3 to 5 too. For those of you who feel it is getting too technical, you may move on to the next paragraph. For the rest this section deserves a second read and careful critical scrutiny.]

### Towards a comprehensive definition of consciousness

#### So what do we do next?

Now that we have an understanding of consciousness, minus the clutter, the best next move to make in this direction, even if to the consternation of many, would be to try and evolve a consensus on a definition of consciousness, keeping in mind the varied interpretations of the stakeholders involved.

#### Working definition of consciousness

As a step in that direction, based on the discussion we have had till now, we may present here a working definition of consciousness, to be worked over and modified, as necessary.

Consciousness is a tetrad of brain functions [CT]. It consists of the following:

1. A default consciousness, which is a primary default state differentiating the living from the dead and the non-living. Primary default state means without this, no other consciousness state can result.
2. Consciousness also consists of a state of aware consciousness which includes the different stages from awake to sleep, including drowsy, dreamy, non-dreamy etc, as also altered states like delirious, comatose, illusionary, hallucinating etc. This is the 2^nd^ or secondary default state on which rest states 3 and 4. Secondary default state means without this, states 3 and 4 cannot result.
3. Consciousness is also a state of operational consciousness wherein all observable/unobservable brain operations like cognition, perception, emotion, conation etc result. Self consciousness, or self-identity, is itself a form of cognition, and therefore a part of operational consciousness. This is the 3^rd^ or tertiary default state, which means without this state, state 4 cannot result.
4. Consciousness is, finally, also a state of exalted consciousness wherein the brain establishes a communion with the ‘inner self,’ the divine, God, soul etc. It does this through some activities of operational consciousness which are furthered and others suppressed/ignored, e.g., cognitive processes like meditation, introspection, focussed concentration, or a mixture of these and related techniques are furthered, while sensory perceptions and emotions etc are ignored or suppressed. This consciousness is the final default state needed for achieving liberation, *mok*
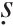
*a* etc.

The *Mens Sana Monographs* would be most pleased to help evolve a comprehensive definition of consciousness in its future issues, taking into consideration the consensus of all stake-holders involved. The above formulation could, hopefully, serve as a starting point.

## II. 4. The lattice of mental operations: Or, how do mental operations result?

One other contentious issue, which must concern us here, is detailing the relationship between observable mental operations and related subtle brain physical activity. In other words, understanding how do mental operations result.

**Figure 6:**
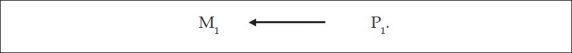
Any mental operation M_1_ is the result of a physical brain activity P_1_

Now that we understand that the brain gives rise to the mind, we will also understand that every mental operation or phenomenon [M_1_] is the result of physical changes in the brain [P_1_], which are due to intraneuronal or interneuronal activity, which latter includes neurotransmission and neurotransmitter activity at the synapses. For example, when one thinks of a mental operation, M_1,_ there is a physical change in the brain that brings this about, P_1_ [Figure 6].

**Figure 7:**
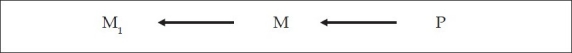
An earlier mental operation M, itself caused by an earlier physical brain activity [P], causes the mental operation M_1_.

**Figure 8:**
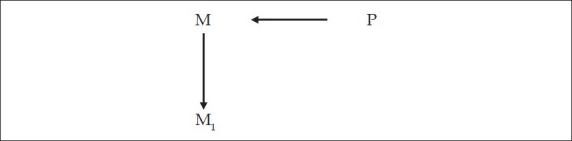
Same as Figure 7. An earlier mental operation M, itself caused by an earlier physical brain activity [P], causes the mental operation M_1_.

**Figure 9:**
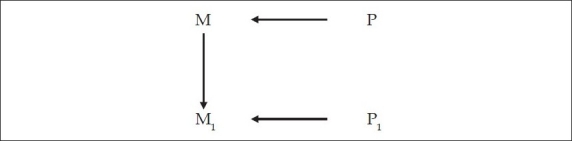
An earlier physical brain activity, P, causes an earlier mental activity M, which causes the present mental activity M_1_ which itself is caused by a physical brain activity P_1_.

But the initiator of this mental activity [M_1_] is an earlier mental operation [M] which itself is the result of a physical change in the brain [P] [Figure 7]. Which means, before one carries out the mental operation of thinking about something, say consciousness, one *decides* to think about it [M], which decision is the result of a physical change in the brain [P]. Or, to say the same thing [see Figure 8]:

But, we already said that P_1_ → M_1_. Thus, we can say, combining Figures 6, and 8, that P causes M, which causes M_1_, which itself is caused by P_1_ [Figure 9]. In our earlier example, one decides to think about consciousness [M] which brings about a thought about consciousness [M_1_], but both these are brought about by their respective antecedent physical changes in brain neurochemistry [P → M, and P_1_ → M_1_].

**Figure 10:**
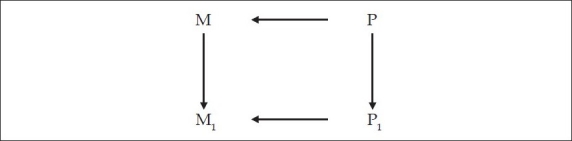
To any mental event M_1_, there is a preceding brain physical activity P_1_, and a preceding mental activity M, which itself is preceded by a brain physical activity P. M → M_1_ is observable mental phenomena, studied by psychologists, neuropsychiatrists, other neuroscientists and philosophers. P → P_1_ is causative subtle physical brain activity, studied by neurobiologists and neuroimaging studies. It is necessary to combine both to get a comprehensive picture.

But we must also note that while M → M_1_, P → P_1_. So, the better way of looking at the figure is as in Figure 10.

Which means that to any mental event M_1_, there is a preceding physical activity P_1_, and a preceding mental activity M, which itself is preceded by a physical activity P.

What is obvious at the gross level is this progression from M → M_1_. This is of interest to philosophers and scientists who work on observable phenomena. What is seen at the subtle level is P → P_1_, which is studied at the neuronal level by neurobiology and neuroimaging [Legend, Figure 10].

**Figure 11:**
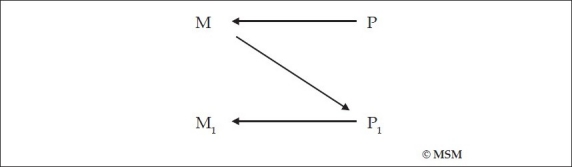
The inverted Z format of mental operations

*But we have still not got the correct picture*. Though we say that a mental operation we observe [M_1_] is the result of an earlier mental operation [M], and the physical operation [P_1_] is the result of an earlier physical operation [P], the relation does not move in parallel; it is more complex [Figure 11]. The initiator of the physical changes in [P_1_] is itself an earlier mental operation [M] which precedes it, which is in the form of a thought, emotion or activity which causes it, which itself is caused due to a physical activity in the brain [P]. That is, P → M → P_1_ → M_1_

Which means, although what we may feel mental operations are like in Figure 10, they are as in Figure 11. Pictorially, an inverted Z.

**Figure 12:**
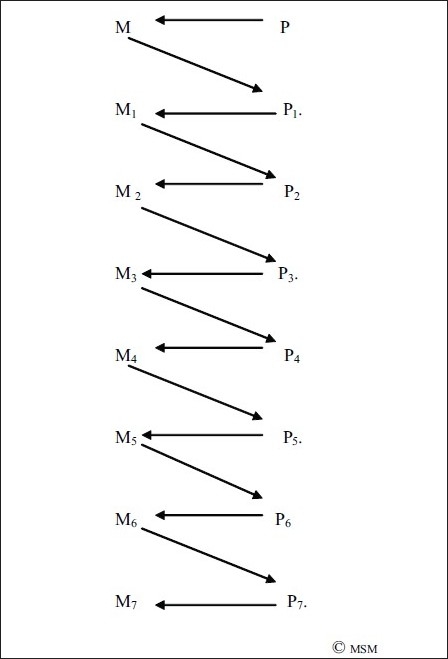
The Inverted Z format of mental operations in a series

This inverted Z is a pictorial representation of the way a mental operation results. This is preceded and succeeded by many other mental operations in one series [Figure 12]. In our earlier example, if one decides to think of consciousness [M] and then presents the first thought [M_1_], followed by its first elaboration [M_2_], followed by successive, say five, elaborations/modifications M_3-7_, we would form a series of inverted Z formation of thought as in Figure 12. [Of course 5 is an arbitrary number.]

Thus results the progression of mental operations from observed mental operation M_1_to observed mental operation M_7_ [Figure 12]_._ And, that is how mental operations in one chain/series continue.

**Figure 13:**
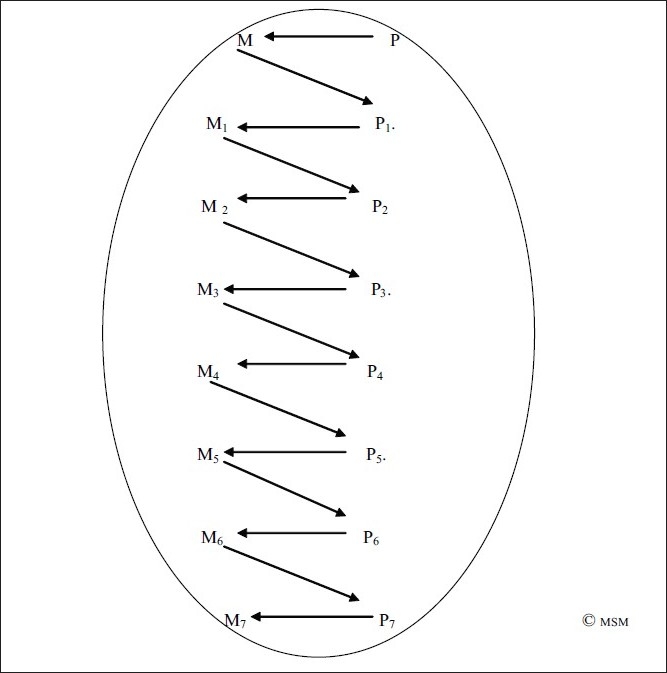
A spiral-oval of inverted Z-shaped mental operations in a series

**Figure 14:**
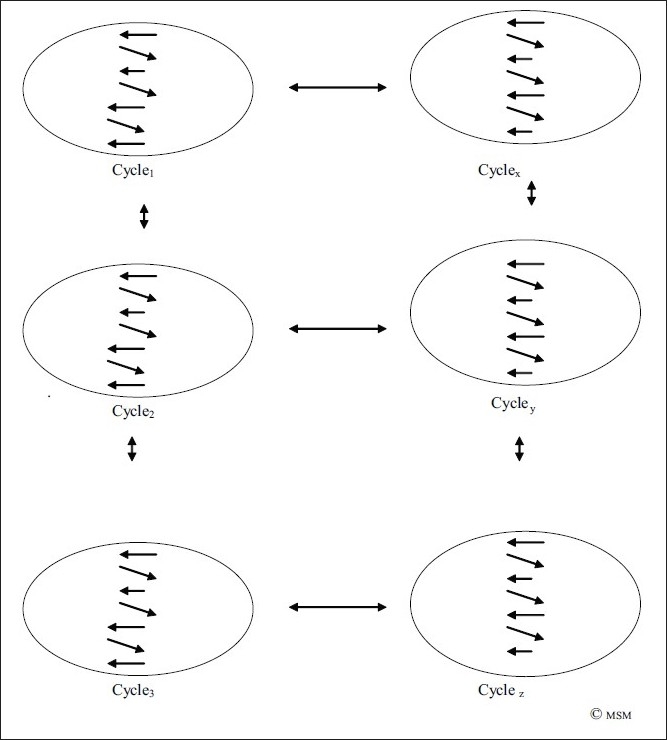
A spiral-oval of thought [Cycle 2] is in dynamic interactions with other spiral-ovals of preceding [C_1_], succeeding [C_3_] and parallel/connected thought spiral-ovals [C_X_, C_Y_] etc, which themselves are in dynamic interaction with each other.

But mental operations do not progress in a linear fashion alone. They are further preceded and succeeded by further such linear progressions. If we consider one such progression pictorially as a spiral-oval [Figure 13], it is connected to and influenced by many such spiral-ovals, which may go on simultaneously [Figure 14].

The more complex is the thought, the greater the cross-linking of such spiral-ovals.

If we call the above as Cycle_2,_ it is preceded by C_1_ and succeeded by many other cycles C_3_, C_4_, C_5_, …… [Figure 14]. It is further influenced by, and in turn influences, many other thought spirals running in parallel, C_x_, C_y_, C_z_ …… For example, while one thinks of consciousness, one may also need to think of answering the ringing phone, and think about what to do about the advance tax to be paid, and also the airline ticket for the upcoming conference, and the car which needs servicing. All these thoughts set up their own cycles, but which interact with the predominant thought at a certain point in time.

**Figure 15:**
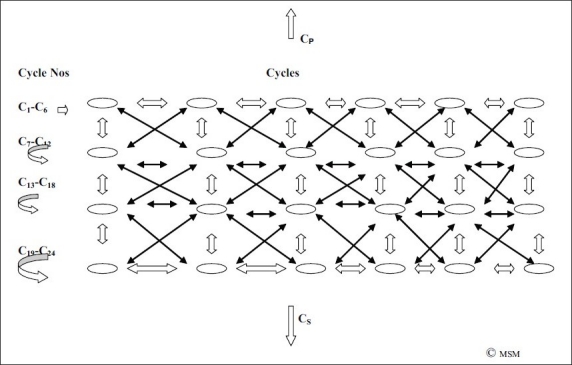
The lattice of mental operations [C_1_–C_24_] in dynamic interaction with each other. The greater the complexity, the greater is the interaction. The complex pattern of thought parallels the complex matrix of neuronal interconnections. Both are criss-crossing and inter-related. There are cycles preceding [C_P_] and succeeding [C_S_] these.

Thus, a huge lattice of thought is formed [Figure 15], a vast matrix of interconnecting inverted Z thought spiral-ovals [what we have called Cycles], which is like a giant web of cross-linked thoughts, thoughts which are also linked with feelings/perceptions/activities which form similar spirals themselves, interacting with-enhancing/modifying/subduing/restricting etc-the expression of a certain thought at a certain point in time. This intricate lattice [Figure 15] only matches the intricate web of the neuronal network that gives rise to it.

As is obvious in Figure 15, the first row of mental operations, C_1_–C_6_, are dynamically inter-related. They are in two-way correspondence. If C_1_ affects C_2_, C_2_ can equally affect C_1_. Similarly, with C_2_ and C_3_, and so on in the first row. But C_1_ is not affected in a linear fashion alone. C_1_ is also affected by C_7_ below, and also by C_8_ in criss-cross. It can also be affected by C_24_ from the other dynamic end. This two-way interconnected correspondence between cycles of thought makes for its complexity. The more complex and inscrutable the thought, the greater are these interconnections. In orderly progression, there are relevant interconnections. In disorganised thought states, bizarre dreams, delirious states, delusions, irrelevant [or seemingly relevant] thought cycles impinge on the mental operation being considered, and give rise to its weirdness and inscrutability. As and when these connections are disentangled or understood by psychotherapy, meditation, introspection, self-knowledge etc, these interconnections are understood, disentangling can occur, and the mental operation can be retrieved from its disorganised and inscrutable state. That is what Freud, for example, tried to do when seemingly weird, or innocuous but disturbing, ideas in the conscious were analysed and the ‘unconscious’ mechanisms that made them weird, or innocuous but disturbing, were disentangled from their disturbing impingings, and thus allowed to manifest in their clear state, free from the envelope of past thoughts and painful emotive underpinnings.

One such lattice presented here, as C_1_ to C_24_, is preceded and succeeded by many such thought lattices, which include the lattices of other mental operations, including emotions, perceptions, memories, motivations, which have their own lattices, but which are in dynamic interaction with each other, and the present and such other thought lattices.

This, a vast network-lattice of mental operations results, closely parallel to the vast lattice of brain neuronal network on which it is based and from which it springs.

This lattice of mental operations is a product of the structural entity brain, which goes to form a functional matrix called mind, results in what is human experience and is possible because of consciousness, which itself is a product of brain operations and one of the functions subsumed under the entity mind.

Thus, brain, mind, consciousness and human experience are interlinked.

## Concluding remarks–The Goal, And Bridging the Gap [Figure 16]

The attempt in this monograph, and in forthcoming ones, is to bring together scholars and intellectuals from diverse streams and evolve a body of knowledge that will further our quest in this intriguing, but still largely inscrutable, area of philosophical/scientific enquiry.

**Figure 16:**
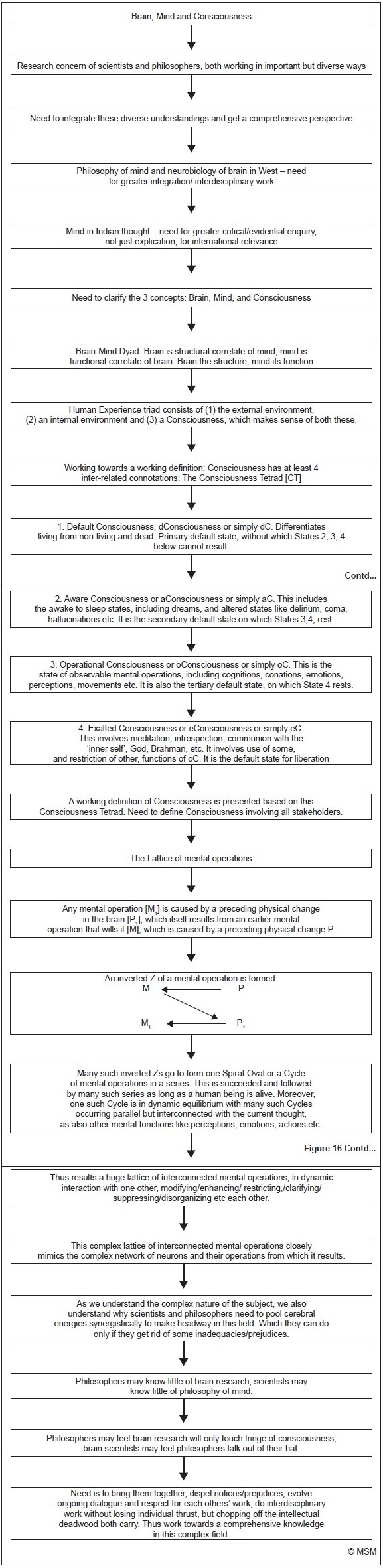
Flowchart of paper

### Why has this not occurred till now?

It is not that it has not, but the attempts have to be furthered with greater vigour. What probably hinders more work in the field is a communication gap, which is the result of a psychological blind spot. Philosophers intimately connected with study of mind and consciousness may know little of brain research. Scientists intimately connected with study of structure and function of the brain may know little about the various theories of mind and consciousness that have engaged philosophers down the centuries, and even actively engages them today. The reasons for this are (1) Both use the same concepts, e.g. ‘mind,’ ‘consciousness’ etc, but speak very different languages technically; and (2) Both make feeble, if any, attempts to make the other understand, or themselves understand what the other has to say–either because of sheer intellectual laziness, or a fear they may have to revise/forsake long-held and life-long commitments to concepts and theories and experience a discomforting intellectual déjà vu.

But we must persist, nevertheless, having faith in the resilience of individuals and the worthiness of the enterprise. The attempt to bring the two bodies of researchers together to evolve a corpus of knowledge that will be mutually beneficial and, hopefully, more than the sum of its parts, needs to be furthered and cannot be forsaken, whatever the obstacles.

The possibility of opening up new areas of research and throwing up new questions for future research, as well as helping contemporary researchers reorient/rethink their present positions/convictions as a result of such interdisciplinary approach is an exciting possibility.

Hence, this monograph.

Critiques and reviews of established positions and theories are welcome, but original contributions are equally to be encouraged. A close look at Indian concepts of mind and consciousness is equally important. We expect this issue and subsequent work in this series to do both.

Before this can be done, and even while doing them, there are some strongly felt but unmouthed convictions that need to be understood as biases/prejudices and disposed of.

What are they?

Often, philosophers harbour a notion that brain research will only touch the fringe of our understanding of mind and consciousness. And often, brain scientists believe that philosophers indulge in speculation devoid of empirical evidence and hence ‘talk out of their hat.’

What, then, need be the attempt?

The attempt must be to bring the two together, which will hopefully dispel these notions and prejudices, and promote much needed respect for each other, and a dialogue and serious study of each others’ work. This will add incrementally to the body of knowledge in the respective fields without they losing their individual thrust. It will also result in a surge in interdisciplinary studies, which can become personal eye openers for individual researchers.

Hence, also this monograph.

## Post-script

### A parting thought: Why at all should philosophers study the brain? and scientists study philosophy of mind?

These questions can be legitimately asked because:

1. Philosophers have managed to give the most intricate theories about mind and consciousness without studying the structure of the brain;
2. Scientists have managed to study the structure of the brain in the most intricate detail without having even a passing acquaintance with the philosophy of mind.

Therefore, would it not be better if both continue to do what they do best? And those who are interested in integrating what they do, can do the integration, and satisfy their curiosity? Why at all try to involve everyone in the enterprise?

Yes, indeed, both should try to do what they do best, and no one prevents them from so doing. That is not the purpose of integrated effort at all. The purpose is to do something when we now realise that their best is not adequate enough; that though they work on similar topics, a clear picture has evaded both, probably because their efforts have been piece-meal and partial. The philosopher is deprived of the experimental method; the scientist is deprived of the methods of reflection. It is like the lame who cannot walk, and the blind who cannot see. But, if both get together, and the lame got on to the shoulders of the blind, the journey could be successfully completed. Of course provided we accept who is lame and who blind. Philosophy can supply the vision, science must supply the limbs. The situation could be remedied only if they synergised efforts, realising their strengths and the stakes for which they play.

There is also another reason. Both philosophers and scientists have a huge mass of theories and data on these topics, and none is ready to, or need, relinquish efforts. This means that no one can any longer afford to neglect the output of the other, for both are equal stakeholders in the enterprise.

Hence, any philosopher who tries to get a comprehensive view of, or tries to give a comprehensive theory of, mind and consciousness today simply cannot afford to neglect the mass of knowledge available from science about these categories from brain research.

A similar situation obtains for scientists and neuroscientists about philosophy of mind. Any scientist who tries to get a comprehensive view of, or attempts to give a comprehensive theory of, mind and consciousness cannot afford to neglect the huge mass of reflections on the topic down the centuries by philosophers of mind, which continues even today.

The neuroscientist, and scientist-philosopher, is in the best position to remedy matters, provided he does not have an aversion for, or harbour deep-rooted prejudices against, either empirical science or reflective philosophy, and is ready to sharpen his skills to do both. Easier said than done.

The task that beckons is to evolve such a comprehensive knowledge, and theory, as will settle, once and for all, all contentious issues about this topic that has intrigued both philosophers and scientists down the centuries. The stakes are large, and hence the effort required has to be equally large to match the task.

### Take home message

There is a need to carefully study the work of diverse appearing branches like philosophy, cognitive neurosciences and biology to get a comprehensive grasp over topics like mind, brain and consciousness.

It is also necessary to define the brain-mind relationship, and to understand how human experience and mental operations result. And also develop a comprehensive definition of consciousness, keeping in mind the views of the different stakeholders involved.

Also needed is a careful understanding of the Indian concept of mind and consciousness.

Finally, it is necessary to forward interdisciplinary work between scientists and philosophers in this field, of course with the caveat that (1) it is without they losing their individual thrusts; (2) without stunting the special contributions of their respective disciplines; and (3) also without stopping their own special contributions in their respective disciplines.

## Questions that this Paper Raises

1. Is it possible to involve all stakeholders to evolve a comprehensive definition of consciousness? Is it at all necessary?
2. Integration of approaches is fine in principle, but will it not give rise to decrease in thrust of the respective fields?
3. Why is it at all necessary to critically evaluate Indian thought when its explication itself is being neglected today? Why not make a sincere and strong attempt to understand what the ancient masters did? Why go for half-baked and incomplete theories, which Westerners keep producing, and revising, when the great Indian masters of yore have already made a complete and systematic delineation?
4. Will philosophers and scientists ever revise their deep-seated opinions and prejudices against each others’ work and approaches? Why not allow them to work based on their strengths and basic assumptions?
5. Mind and brain need to be understood in a proper perspective is fine as a thought. But, the concept mind has already been developed and written about for centuries by philosophers both of the east and the west. How can we just discard all their work by formulating a relationship that negates the entity status to the mind?
6. Why can scientists not let the philosophers carry on with their concept of mind and consciousness, and they carry on with their research on the brain? Why should they try to hijack the agenda and usurp all the space in this field by negating the contributions of mind-body and other philosophical theories to the field?
7. The Consciousness Tetrad needs further delineation. Does it seek to replace the different formulations about consciousness in existence today?
8. Consciousness as a metaphysical concept does not lend itself to scientific enquiry. Why not, then, let it be so? Why should science try to step into philosophy’s domain? Why not live and let live? Allow each to grow in its own way, with their own assumptions, and within its own domain and sphere of influence?
9. The lattice of mental operations must provide for the exact relationship between physical brain operations and consequent brain functions. How do we accept what come first?

## About the Author

Ajai R. Singh, M.D., is a Psychiatrist and Editor, Mens Sana Monographs, (http://www.msmonographs.org). He has written extensively on issues related to psychiatry, philosophy, bioethical issues, medicine and the pharmaceutical industry. © MSM

## About the Author

Shakuntala A. Singh, Ph.D., is Principal, Reader and Head, Dept of Philosophy, K.G. Joshi College of Arts and N.G. Bedekar College of Commerce. She is also Deputy Editor of MSM. Her areas of interest are Indian philosophy, bio-ethics, logic and the philosophy of science. © MSM
